# High rates of transmitted NNRTI resistance among persons with acute HIV infection in Malawi: implications for first-line dolutegravir scale-up

**DOI:** 10.1186/s12981-019-0220-8

**Published:** 2019-02-22

**Authors:** Sarah E. Rutstein, Jane S. Chen, Julie A. E. Nelson, Samuel Phiri, William C. Miller, Mina C. Hosseinipour

**Affiliations:** 1 Division of Infectious Diseases, University of North Carolina, Chapel Hill, NC USA; 2 Department of Epidemiology, University of North Carolina, Chapel Hill, NC USA; 3 Department of Microbiology and Immunology, University of North Carolina, Chapel Hill, NC USA; 4 Lighthouse Trust, Lilongwe, Malawi; 5 Department of Epidemiology, Ohio State University, Columbus, OH USA; 6 UNC Project, Lilongwe, Malawi

**Keywords:** Antiretroviral resistance, Acute HIV infection, Malawi, Dolutegravir

## Abstract

High rates of non-nucleoside reverse transcriptase inhibitors (NNRTI) resistance was a key consideration in the WHO policies transitioning first-line regimens to include integrase inhibitors (dolutegravir [DTG]). However, recent data suggests a relationship between DTG and neural tube defects among women exposed during conception, giving providers and policymakers pause regarding the planned regimen changes. We examined HIV drug resistance among a cohort of 46 acutely infected persons in Malawi. Our data demonstrates high levels of transmitted resistance, 11% using standard resistance surveillance mutations and 20% when additional NNRTI polymorphisms that may affect treatment response are included. High resistance rates in this treatment-naïve patient population reinforces the critical nature of DTG-based options in the context of public-health driven treatment programs.

## Background

Current WHO-recommended first-line antiretroviral therapy (ART) regimens include non-nucleoside reverse transcriptase inhibitor (NNRTI) efavirenz in addition to two nucleoside analogue reverse transcriptase inhibitors [1]. However, increasing prevalence of NNRTI resistance among those initiating ART have raised concerns regarding the public health implications of efavirenz-based first-line regimens [2–6], as people with NNRTI-resistance are up to three times as likely to fail first-line therapy [7–11]. In response to escalating resistance, alternative first-line regimens may be critical to preserve morbidity and mortality benefits of early ART, as well as delay or avoid costly switch to second-line ART.

Dolutegravir (DTG) has been promoted as an alternative first-line ART backbone, with potential efficacy and safety advantages over efavirenz and a very high barrier to resistance [12–14]. However, the logistical, cost, and clinical merits of such a policy shift depend directly on the underlying NNRTI-resistance patterns and prevalence. Furthermore, recent observational data from Botswana suggests a potential relationship between DTG and neural tube defects among women on DTG at time of conception has given many providers and policymakers pause regarding universal transitioning to a DTG-based backbone [15].

Here, we summarize NNRTI resistance patterns observed among treatment-naïve persons in Malawi identified with acute HIV infection (AHI). We include all mutation polymorphisms that potentially affect treatment response, as well as summarize mutations according to the WHO surveillance of drug resistance mutations list—widely accepted as those mutations most likely to represent transmitted drug resistance [16]. Our findings inform the ongoing conversation regarding risks and benefits of transitioning from efavirenz-based first-line to DTG-based first-line treatment regimens.

## Analytical methods

We analyzed resistance patterns from peripheral blood specimens collected at time of enrollment among participants in a pilot study evaluating behavioral and biomedical interventions for persons with AHI in Lilongwe, Malawi. Participants were screened and enrolled into the study between June 2012 and January 2014. Description of screening and study protocols are described elsewhere (clinicaltrials.gov #NCT01450189) [17, 18]. AHI was defined as detectable HIV RNA with discordant or negative rapid HIV antibody tests. Where specimen volume allowed (39/46 specimens), repeat rapid antibody testing was conducted using rapid diagnostic assays (Determine™ HIV 1/2 Antibody [Abbott Diagnostics, Illinois US] and Uni-Gold Recombigen^®^ HIV 1/2 [Trinity Biotech, Ireland]) to confirm antibody negative/discordancy. We did not screen for receipt of pre or post-exposure prophylaxis; PrEP was not available in Malawi at the time of the study and although remote exposure to PEP was possible, PEP exposure since the very recent infection acquisition was considered unlikely given that screening represented the first presentation to care for this cohort of acutely infected persons. Additional eligibility criteria included: above definition of AHI within 21 days of enrollment, age ≥ 18 years, and intention to remain in the Lilongwe area for the duration of study follow-up (52 weeks).

Integrase and reverse transcriptase (RT) genotypic resistance testing was performed for all participants using specimens collected at time of enrollment, which was on average 8 days after screening. All genotypic resistance testing was conducted on stored specimens after completion of study activities and thus was not available for real-time patient management. Reverse transcriptase population sequencing to determine resistance was performed as described previously [19]. After alignment using Sequencher (Gene Codes), resistance mutations were classified based on information in the Stanford University Drug Resistance Database (http://hivdb.stanford.edu/index.html). We present both the full complement of resistance mutations as well as the resistance rates as limited to those identified by the WHO surveillance of drug resistance mutations list (SDRM) [16]. Sample integrity was checked by aligning the sequences using CLC Sequence Viewer (CLC bio A/S). Ninety-five percent confidence intervals (CI) were calculated using exact methods in Stata (version 14.1, College Station, TX).

The National Health Sciences Research Committee of Malawi, the Malawi Medicines and Poisons Board, the Biomedical Institutional Review Board at University of North Carolina, Chapel Hill, and the National Institute of Allergy and Infectious Diseases’ Prevention Science Review Committee approved the procedures for this study. All study participants provided written informed consent in the local language or English, if preferred.

## Results and discussion

Among 59 persons identified with AHI, 46 were enrolled into the study [20]. Of these 46 participants, 45 had resistance testing; nine (20%; 95% CI 10–35%) had at least one NNRTI resistance mutation detected (Table 1) and three had NRTI mutations detected (Table 2). There were no integrase mutations detected. Limiting likely transmitted drug resistance classification to those with mutations appearing in the SDRM list, five (11%; 95% CI 4–24%) had NNRTI resistance; the most frequent mutation was K103N, detected in four participants with any NNRTI mutations. The second most common mutation was E138A (3/9: 33%), a mutation that is not on the SDRM but which nonetheless may be clinically significant in its effect on treatment response.

**Table 1:** Demographics and resistance outcomes

	Total (n = 46) N (%)	No resistance (n = 36) N (%)	Any resistance (n = 9) N (%)
Age			
18–24	19 (41)	14 (78)	4 (22)
25–34	20 (43)	15 (75)	5 (25)
35–44	6 (13)	6 (100)	0 (0)
≥ 45	1 (2)	1 (100)	0 (0)
Sex			
Male	28 (61)	21 (78)	6 (22)
Female	18 (39)	15 (83)	3 (17)
Marital status			
Never married	11 (24)		
Married	23 (50)		
Separated/divorced/widowed	12 (26)		
Viral load at screening			
≤ 6 log_10_ copies/mL	24 (53)	19 (83)	4 (17)
> 6 log_10_ copies/mL	21 (47)	16 (76)	5 (24)
NNRTI resistance mutations frequency^a^			
A98G	2 (4.4)		
E138A	3 (6.7)		
K101E	1 (2.2)		
K103N	4 (8.9)		
K103Q	1 (2.2)		
V179E	1 (2.2)		
V90I	1 (2.2)		
Y181C	1 (2.2)		

*NNRTI* non-nucleoside reverse transcriptase inhibitors

^a^Among 45 patients assessed for resistance at time of study entry

**Table 2:** HIV drug resistance mutation profiles

Profile #	NNRTI mutation	NRTI mutation
1	A98G^a^, K103N	M184V, T215F
2	E138A^a^	
3	E138A^a^	
4	E138A^a^, V179E^a^	
5	K103N	
6	K103N	
7	K103N	
8	K103Q^a^	
9	V90I^a^, A98G^a^, K101E, Y181C	T69N
10	None	K219R

*NNRTI* non-nucleoside reverse transcriptase inhibitors, *NRTI* nucleoside reverse transcriptase inhibitors, *WHO* World Health Organization

^a^Designated mutations are not on the WHO surveillance of drug resistance mutations list and thus may be less likely to represent transmitted drug resistance

Increasing levels of ART resistance are jeopardizing the success of ART scale-up, and specifically may compromise the efficacy and effectiveness of existing first-line, efavirenz-based ART regimens. WHO surveillance demonstrates steadily increasing resistance since 2001, particularly in southern and eastern Africa [2, 6]. Pre-treatment drug resistance has previously been estimated upwards of 10% [6]; in our study, among persons with AHI, transmitted drug resistance was identified in 11% (according to SDRM definitions) and 20% of patients at least one NNRTI mutation. Observed rates are similar to those in recent WHO reports from Malawi, where 4/26 (15%) of ARV drug-naïve persons had NNRTI mutations [6]. High resistance rates support the urgency of the DTG transition—a likely cost-effective programmatic shift of first-line ART regimen [21].

Vigilance is needed in monitoring response to therapy and any possible AE related to initiation of DTG. Ongoing evaluations include prospective clinical trials and observational studies focusing on several key populations (pregnant women, HIV–TB co-infection) [22]. Nonetheless, improved safety outcomes profiles, higher barriers to resistance, more favorable clinical tolerability, and cost-effectiveness modeling all suggest that DTG is a preferred first-line agent compared to efavirenz. Initiation of DTG in women of childbearing potential should be pursued cautiously, and with an informed provider and patient-population [15]. Our data demonstrates high levels of transmitted NNRTI resistance in Malawi, compromising the effectiveness EFV-based regimens. These data underscores the urgency of ongoing evaluations of the safest means by which to transition treatment initiation to DTG-based options. Persons with AHI represent a unique population for evaluation of transmitted drug resistance and similar evaluations may be warranted in other LMIC to better clarify implications of EFV-backbone as first-line therapy.

## Abbreviations

AE: adverse event
AHI: acute HIV infection
ART: antiretroviral therapy
DTG: Dolutegravir
EFV: efavirenz
LMIC: low- and middle-income countries
NNRTI: non-nucleoside reverse transcriptase inhibitors
NRTI: nucleoside reverse transcriptase inhibitors
RT: reverse transcriptase
SDRM: surveillance of drug resistance mutations
WHO: World Health Organization
